# Double or nothing: red flag symptoms of critical carotid stenosis, a case report

**DOI:** 10.1186/s12883-017-0849-7

**Published:** 2017-04-05

**Authors:** José Carlos Morán-Sánchez, Irene Gómez-Estévez, Yasmina El Berdei, José C. Gómez-Sánchez, María E. Ramos-Araque

**Affiliations:** 1 Department of Neurology, University Hospital of Salamanca, Paseo de San Vicente 58-182, 37007 Salamanca, Spain; 2 Institute of Biomedical Research of Salamanca, University Hospital of Salamanca, Salamanca, Spain

**Keywords:** Critical carotid stenosis, Cervical radiotherapy, Ocular motor palsy, Amaurosis fugax, Double or nothing syndrome, Case report

## Abstract

**Background:**

Detailed knowledge of every possible manifestation of Internal Carotid Artery (ICA) disease is important. For improving detection and a timely adoption of secondary prevention procedures or treatments. Transient oculomotor nerve palsies have been described associated with stenosis or occlusion of the ICA.

**Case presentation:**

We described a patient that develop a sequential combination of transient monocular loss of vision followed by binocular diplopia secondary to an unstable atherosclerotic preocclusive stenosis of an internal carotid artery previously treated with radiotherapy.

**Conclusions:**

The peculiar sequence of transient monocular vision that give rise later into a transient binocular diplopia (double or nothing) should be kept in mind as a possible manifestation of critical stenosis of ICA.

## Background

Appropriate knowledge of every possible clinical expression of extracranial carotid disease is crucial. Accurate identification of symptomatic carotid disease is necessary for a timely adoption of secondary prevention procedures or treatments. When symptomatic, internal carotid artery (ICA) stenosis usually gives rise to brain ischemic disease, ocular ischemic disease or both. The paradigmatic ocular manifestation consists on the sudden and transitory loss of monocular vision because retinal ischemia [1]. But, in the past few years, several clinical cases of binocular diplopia caused by oculomotor nerve palsies have been described associated with stenosis or occlusion of the ICA [2, 3]. The majority of these cases have been reported on young patients with arterial dissection and few cases in patients after radiation induced atherosclerosis [4, 5].

Detailed knowledge of the clinical characteristics of those cases with diplopia as a manifestation of ICA disease would improve their detection. We describe a patient with a sequential combination of transient loss of monocular vision immediately followed by binocular diplopia, as an atypical form of presentation of ICA stenosis, which could be named “double or nothing”.

## Case presentation

A 57-year-old man experienced acute onset of transient monocular painless visual loss in his left eye that lasted for over ten minutes. After occluding alternatively only one eye it was established that it was a complete, left, unilateral amaurosis. The recovery was slow, the patient started to see from the bottom up, “as if a blind were rising front of the eye”. Afterwards he suffered diplopia and ipsilateral eyelid ptosis for several hours. The next morning he was already asymptomatic. Past medical history included smoking (20 packs cigarretes a year for 20 years) and a larynx epidermoid carcinoma that had been treated with surgery and radiotherapy ten years before. There was also past history of dyslipidemia and arterial hypertension treated with statins and a renin-angiotensin inhibitor. Neurologic examination (performed after amaurosis recovery and while diplopia) revealed on the left eye: eyelid ptosis and ofthalmoparesis of extrinsic muscles: inferior rectus, superior rectus and internal lateral rectus with intrinsic muscles respected. Pupils were equal in size with conserved direct photomotor reflex and a consensual response. General examination showed first-degree obesity and tracheotomy. Urine and blood analysis (including autoimmune analysis, and serology for neurotropic infectious agents -Treponema Pallidum, Brucella Spp, Borrelia Burgdorferi, Cytomegalovirus, Epstein Barr, Herpex simplex, Varicella Zoster- and acute phase reactants) were normal with the exception of a tiny rise in High density lipoprotein (HDL) cholesterol. Findings on head computed tomography (CT) 3 h after the onset of symptoms were normal. A pattern compatible with a critical occlusion/obstruction of the left ICA was revealed on ultrasonographic study of the supra-aortic trunks: the peak systolic velocity was undetectable, the lumen of the artery was markedly narrowed at colour and power Doppler ultrasound. The atherosclerotic plaque was heterogeneous, hypoechoic, the surface was smooth.

**Fig. 1:**
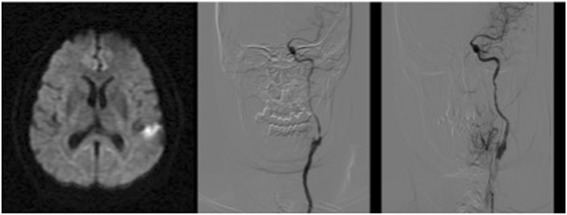
Diffusion MR Image showing hemispheric left focal hyperintensity area after the symptomatology and angiographic images showing left extracranial ICA critical stenosis before and after endovascular recanalization

**Fig. 2:**
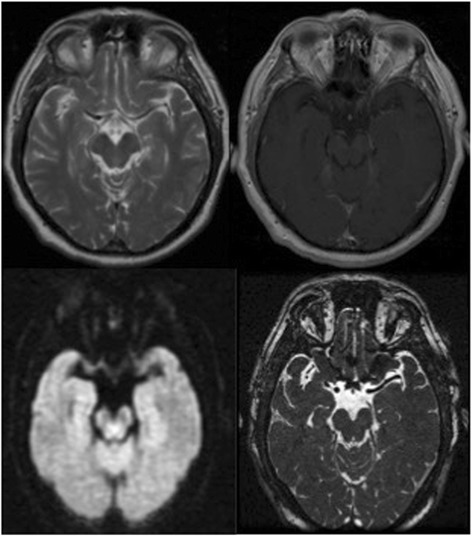
MR cuts including the midbrain and orbit. Sequences: T2, T1 + contrast, Difusion, FIESTA

**Fig. 3:**
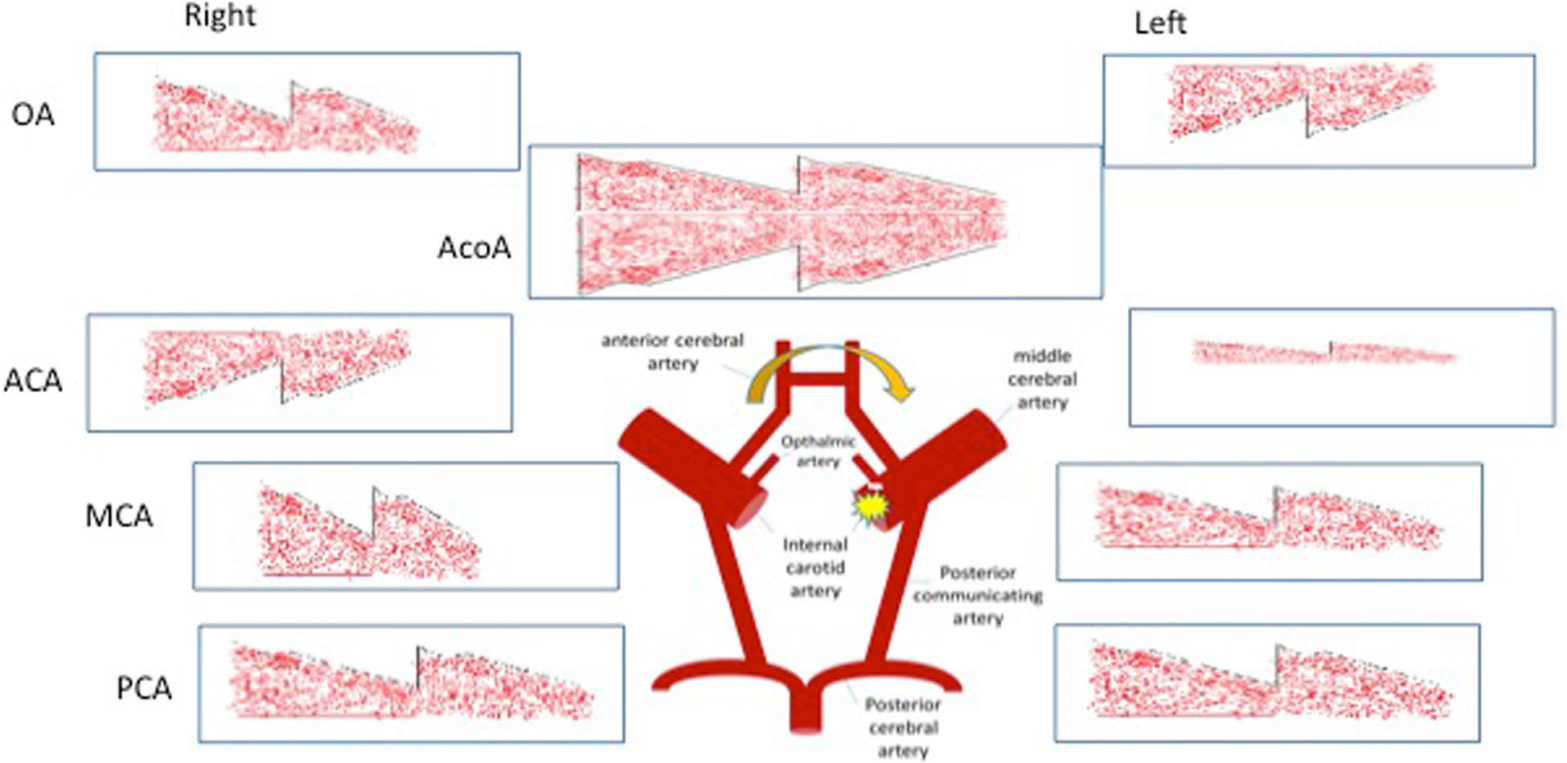
The left ophthalmic artery blood supply was obtained through the anterior communicating artery of the circle of Willis and flow reversal of the left anterior cerebral artery. OA: opthalmic artery. AcoA: Anteior communicating artery. ACA: anterior cerebral artery. MAC: middle cerebral artery. PCA: posterior cerebral artery

Cranial magnetic resonance (MR) and MR angiography of the supra-aortic trunks showed an acute infarct on the left lentiform nucleus (Fig. 1), as well as a 95% and 60% stenosis of the left and right ICA respectively. MR cuts including the midbrain and orbit are shown in Fig. 2. These carotid stenosis were confirmed on digital subtraction angiography (DSA), an occlusion was observed in the ICA from its origin. The circulation of the left internal carotid artery came from collateral flow through the anterior communicating artery. The left ophthalmic artery blood supply was obtained through the anterior communicating artery of the circle of Willis and flow reversal of the left anterior cerebral artery (Fig. 3). The ophthalmic artery is a branch of the internal carotid artery. Carotid balloon angioplasty and the placement of an endoprosthesis with embolic protection system (The Carotid Wallstent Monorail Endoprosthesis with the Boston Scientific embolic protection system type FilterWire EZ™, and diameter guidewire of 0.014 in - 0.36 mm-) on the left ICA were performed without complications (Fig. 1). During 8 weeks after the surgery the patient was under double antiplatelet regimen with clopidogrel 75 mg/day and acetylsalicylic acid 300 mg/ day. Thereafter he has been under single treatment with acetylsalicylic acid 300 mg/day. After a 3 year follow up, the patient has not suffered any new symptom. Control sonographic studies have confirmed the permeability of the operated artery.

## Discussion and conclusion

This case shows the unique sequence of transient monocular visual loss with, thereafter, transient third nerve palsy, in a patient with a complicated atherosclerotic plaque on ipsilateral extracranial ICA and significant atherosclerosis in other supra-aortic trunks.

Amaurosis fugax and diplopia were caused by retinal and third cranial nerve ischemia respectively, both on the left side.

Amaurosis fugax is a transitory loss of the monocular vision due to ischemia on the territory irrigated by the ophthalmic artery. Theoretically complete acute palpebral ptosis could trick the patient into thinking that he had lost his vision. But a quick examination by either the patient or an external subject can help discern which the cause of the loss of vision is.

The mechanism for amaurosis fugax could have been arterial-arterial embolism coming from an extracranial ICA mural thrombus and/or atheromatous material, explaining to the silent ipsilateral cerebral infarction on MR. The associated generalized arteriopathy of the other supra-aortic trunks could have determined a higher drop in the retinal-choroidal blood flow.

Ischemic neuropathy of the Third cranial nerve due to extracranial carotid arteriopathy is an uncommon occurrence.

Third cranial nerve vasa nervorum come from a complex network of multiple small branches. This anastomotic plexus is supplied in its anterior segments, from the maxillary and ophthalmic arteries. Posteriorly, posterior cerebral and cavernous portion of internal carotid branches, give rise other vessels for the network [2]. This pattern of blood supply, coming from different arterial territories, could explain why oculomotor palsy is very uncommon, even transiently, after carotid occlussions or carotid ligation during treatment of aneruysms [4] since occlusion of several components of the nerves nutrient supply is probably required to produce significant ischemia [6–8].

The possible combination of fibrotic changes in vessel layers and radiation-induced atherosclerosis involving not only ICA, but also other facial and supra-aortic vessels, could had determined a basal hypoperfusion situation in the third cranial nerve. The intracranial internal carotid arterial-arterial embolisms caused by extra cranial plaque vulnerability could determine the acute claudication of the vascular feeding of the third nerve and the transient ischemic neuropathy [3, 5].

The present case shows a sequence of clinical events that has not been previously described. The phenomenon that we have named “double or nothing” would consist on the complete loss of monocular vision that lasted for minutes that give rise later into a transient binocular diplopia. This peculiar sequence of clinical events should be kept in mind as a possible manifestation of critical stenosis of ICA, particularly if the blood flow of the arteries that form the supra-aortic trunks are compromised due to a prior cervical irradiation.

## Abbreviations

CT: Computed Tomography
DSA: Digital subtraction angiography
HDL: High density lipoprotein
ICA: Internal Carotid Artery
MR: Magnetic Resonance
